# Supplementary material for: Effectiveness of interventions for dementia in low- and middle-income countries: protocol for a systematic review, pairwise and network meta-analysis
Source: BMJ Open. 2019 Jun 19;9(6):e027851. doi: 10.1136/bmjopen-2018-027851 (PMC6588974; doi:10.1136/bmjopen-2018-027851)
Supplement: Supplementary data [file bmjopen-2018-027851supp001.pdf]

<u>Online supplementary material</u>

Effectiveness of interventions for dementia in low- and middle-income countries: Protocol for a systematic review, pairwise and network meta-analysis

Maximilian Salcher-Konrad, Huseyin Naci, David McDaid, Suvarna Alladi, Déborah Oliveira, Andra Fry, Shereen Hussein, Martin Knapp, Christine Musyim, David M. Ndetei, Mariana Lopez-Ortega, Adelina Comas-Herrera

Supplementary table: Draft outcome categories

Types of outcomes	Outcomes relevant for: Person living with dementia	Carer	Care system/society
Diagnosis	X		X
Clinical outcomes			
- Cognition	X		
- Depression and anxiety	X		
- Behaviour	X		
- Physical health	X		
Quality of life and functioning			
- Quality of life of person with dementia	X		
- Activities of daily living/IADL	X		
- Social and role functioning	X		
Carer outcomes			
- Quality of life of carer		X	
- Carer burden		X	
- Mental health of carer		X	
- Carer economic outcomes		X	
- Carer professional development		X	
- Carer retention		X	
Care provided			
- Satisfaction with care	X	X	X
- Care setting (institutionalisation)	X		
- Appropriateness and quality of care (e.g. use of feeding tubes)	X		X
Economic outcomes			
- Service use and cost reductions			X
- Out-of-pocket payments	X	X	
- Opportunity costs	X	X	X
End-of-life			
- Place of death	X		
- Decision-making	X		
